# Indivíduos Doentes e Populações Doentes

**DOI:** 10.36660/abc.20260436

**Published:** 2026-07-21

**Authors:** Érique José Farias Peixoto de Miranda

**Affiliations:** 1 Instituto Butantan – Ensaios Clínicos São Paulo SP Brasil Instituto Butantan – Ensaios Clínicos, São Paulo, SP – Brasil

**Keywords:** Sistemas de Informação Geográfica, Epidemiologia, Mortalidade, Doença Crônica

O artigo de Lederman et al.^1^ discute uma investigação epidemiológica de mortalidade por doenças dos sistemas circulatório, respiratório e endócrino, além de neoplasias, na Região Metropolitana de São Paulo (RMSP), analisada por geoprocessamento.

É meritório notar o fato de o estudo ter combinado uma base de mais de 1,3 milhão de registros hospitalares provenientes de um hospital terciário com registros de mortalidade estaduais por meio de *linkage* determinístico. Em outras palavras, variáveis idênticas do mesmo paciente presentes nas diferentes bases de dados foram usadas para combiná-las, o que permite acompanhar desfechos em longo prazo de pacientes frequentemente perdidos após a alta hospitalar.

Poucos estudos nacionais dispõem de mais de um milhão de registros clínicos vinculados a dados de mortalidade. Essa escala amplia a robustez descritiva dos achados e reforça o valor dos prontuários eletrônicos como instrumentos epidemiológicos. Além disso, o trabalho evidencia a importância institucional da integração entre hospitais terciários, fundações públicas de dados e sistemas de vigilância em saúde. Por meio da avaliação da distribuição espacial, se pôde compreender evidências de interações entre as doenças crônicas e os fatores sociais, ambientais e estruturais.

Tradicionalmente, os estudos de doenças crônicas se concentram em fatores individuais de risco.^2^ Entretanto, o espaço geográfico não é mero cenário passivo da doença, mas um determinante ativo da saúde. Geoffrey Rose^3^ em seu clássico "*Sick Individuals, Sick Populations*" demonstra a importância da transição da epidemiologia do indivíduo para o território e população, além de enfatizar a doença como produto do contexto social, geografia como marcador de risco coletivo e prevenção populacional em comparação com a prevenção clínica. O estudo^1^ abordou esses pontos, além de posicionar o hospital como observatório populacional.

Embora a RMSP tenha índices de desenvolvimento humano acima da média do Brasil, a região apresenta profunda segregação espacial entre a capital e a periferia, determinando diferenças de renda, de acesso à saúde, de modo que podem explicar a heterogeneidade dos dados de mortalidade obtidos no estudo. Sendo assim, o local de residência sintetiza desigualdades socioeconômicas, disponibilidade de serviços, saneamento básico, exposição ambiental, mobilidade urbana, densidade populacional, qualidade do ar, ruído e acesso ao cuidado especializado.

Sendo assim, interpretamos que mesmo em um contexto de cidades que têm o maior produto interno bruto (PIB) e o maior PIB *per capita* do Brasil, iniquidades sociodemográficas, econômicas e geográficas influenciam, de um modo elegantemente mensurado, os indicadores de mortalidade. Iniciativas de uso de geoprocessamento são potencialmente otimizadoras de recursos públicos em saúde e já têm sido implementadas na Atenção Primária e na Vigilância em Saúde do Sistema Único de Saúde (SUS), permitindo planejamento regional de saúde.

A organização do SUS depende fortemente de mecanismos de regionalização e hierarquização do cuidado. Entretanto, muitas vezes o planejamento assistencial ainda se apoia em indicadores administrativos agregados, incapazes de captar microterritórios de maior vulnerabilidade. Mapas de densidade de doenças, como os apresentados neste trabalho, podem auxiliar gestores na alocação de recursos, definição de linhas de cuidado, expansão de serviços diagnósticos e desenvolvimento de estratégias preventivas focalizadas.

Os autores demonstraram que mortalidade por doenças crônicas apresentam distribuição heterogênea na Grande São Paulo. Alguns achados merecem destaque especial. As maiores densidades de mortalidade por doenças do aparelho circulatório foram observadas em São Paulo, Osasco e Taboão da Serra, enquanto determinadas cidades periféricas exibiram elevadas concentrações de comorbidades cardiovasculares. Esses dados sugerem que a carga cardiovascular não se distribui aleatoriamente, mas acompanha e provavelmente reflete desigualdades urbanas históricas.

É particularmente interessante a observação de elevadas densidades de doenças respiratórias em municípios marcados por intenso fluxo veicular ou concentração industrial, como Osasco e Embu das Artes. Ainda que o estudo não tenha sido desenhado para estabelecer causalidade ambiental, esses achados convergem para a literatura recente sobre poluição atmosférica e doenças cardiopulmonares no Brasil.^4^ A interação entre poluentes ambientais, inflamação sistêmica e eventos cardiovasculares constitui hoje um dos campos mais promissores da Cardiologia Preventiva.^5,6^

O estudo^1^ tem limitações. É unicêntrico, proveniente de hospital altamente especializado, o que produz vieses de encaminhamento. A distribuição espacial observada não representa a prevalência populacional das doenças, mas sim a distribuição dos pacientes que alcançaram um centro terciário de referência. Além disso, não houve padronização por idade, sexo ou condição socioeconômica, fatores importantes na composição dos clusters geográficos identificados.

Outra limitação consiste no uso do endereço residencial como marcador territorial. Em grandes metrópoles, deslocamentos cotidianos relacionados ao trabalho e mobilidade urbana podem modificar as exposições ambientais reais dos indivíduos. Ainda assim, mesmo com essas limitações, o estudo preserva valor exploratório e gerador de hipóteses.

Entretanto, o artigo^1^ também ilustra uma tendência crescente da Medicina contemporânea: a convergência entre *big data*, epidemiologia espacial e inteligência em saúde. Assim, o estudo oferece contribuição original ao ajudar a compreender a geografia das doenças crônicas em uma das maiores regiões metropolitanas do mundo.
