# Supplementary material for: Indivíduos Doentes e Populações Doentes
Source: Arq Bras Cardiol. 2026 Jul 21;123(6):e20260436. [Article in Portuguese] doi: 10.36660/abc.20260436 (PMC13399134; doi:10.36660/abc.20260436)
Supplement: Translation

# Translation: Sick Individuals and Sick Populations

**DOI:** 10.36660/abc.20260436i

**Authors:** Érique José Farias Peixoto de Miranda

**Affiliations:** 1 São Paulo SP Brazil Instituto Butantan – Ensaios Clínicos, São Paulo, SP – Brazil

**Keywords:** Geographic Information Systems, Epidemiology, Mortality, Chronic Disease

The article by Lederman et al.^1^ discusses an epidemiological investigation of mortality from diseases of the circulatory, respiratory, and endocrine systems, as well as neoplasms, in the São Paulo Metropolitan Region (SPMR), analyzed using geoprocessing.

It is noteworthy that the study combined a database of over 1.3 million hospital records from a tertiary hospital with state mortality records through deterministic linkage. In other words, identical variables from the same patient present in the different databases were used to combine them, allowing for the tracking of long-term outcomes of patients frequently lost to care after hospital discharge.

Few national studies have more than one million clinical records linked to mortality data. This scale increases the descriptive robustness of the findings and reinforces the value of electronic health records as epidemiological tools. Furthermore, the work highlights the institutional importance of integration between tertiary hospitals, public data foundations, and health surveillance systems. Through the evaluation of spatial distribution, it was possible to understand evidence of interactions between chronic diseases and social, environmental, and structural factors.

Traditionally, studies of chronic diseases focus on individual risk factors.^2^ However, geographic space is not merely a passive setting for disease, but an active determinant of health. Geoffrey Rose,^3^ in his classic "Sick Individuals, Sick Populations," demonstrates the importance of the transition from individual epidemiology to territory and population epidemiology, in addition to emphasizing disease as a product of social context, geography as a marker of collective risk, and population-based prevention in comparison to clinical prevention. The study^1^ addressed these points, as well as positioning the hospital as a population observatory.

Although the SPMR has Human Development Index scores above the Brazilian average, the region exhibits profound spatial segregation between the capital and the periphery, determining differences in income and access to healthcare, which may explain the heterogeneity of mortality data obtained in the study. Therefore, place of residence synthesizes socioeconomic inequalities, availability of services, basic sanitation, environmental exposure, urban mobility, population density, air quality, noise, and access to specialized care.

Therefore, we interpret that even in a context of cities with the highest gross domestic product (GDP) and the highest GDP per capita in Brazil, sociodemographic, economic, and geographic inequalities influence mortality indicators in an elegantly measurable way. Geoprocessing initiatives are potentially optimizing for public health resources and have already been implemented in Primary Care and Health Surveillance within the Unified Health System (SUS), enabling regional health planning.

The organization of the SUS strongly depends on mechanisms for regionalization and hierarchical organization of care. However, healthcare planning often still relies on aggregated administrative indicators, which are unable to capture micro-territories of greater vulnerability. Disease density maps, such as those presented in this work, can assist managers in allocating resources, defining care pathways, expanding diagnostic services, and developing targeted preventive strategies.

The authors demonstrated that mortality from chronic diseases presents a heterogeneous distribution in Greater São Paulo. Some findings deserve special attention. The highest mortality densities from diseases of the circulatory system were observed in São Paulo, Osasco, and Taboão da Serra, while certain peripheral cities exhibited high concentrations of cardiovascular comorbidities. These data suggest that the cardiovascular burden is not randomly distributed but follows and likely reflects historical urban inequalities.

It is particularly interesting to observe high densities of respiratory diseases in municipalities marked by heavy vehicular traffic or industrial concentration, such as Osasco and Embu das Artes. Although the study was not designed to establish environmental causality, these findings converge with recent literature on air pollution and cardiopulmonary diseases in Brazil.^4^ The interaction between environmental pollutants, systemic inflammation, and cardiovascular events is currently one of the most promising fields in Preventive Cardiology.^5,6^

The study^1^ has limitations. It is a single-center, originating from a highly specialized hospital, which introduces referral biases. The observed spatial distribution does not represent the population prevalence of the diseases, but rather the distribution of patients who reached a tertiary referral center. Furthermore, there was no standardization by age, sex, or socioeconomic status, important factors in the composition of the identified geographic clusters.

Another limitation lies in the use of residential address as a territorial marker. In large metropolises, daily commutes related to work and urban mobility can modify individuals’ actual environmental exposures. Even so, despite these limitations, the study retains exploratory value and generates hypotheses.

However, the article^1^ also illustrates a growing trend in contemporary medicine: the convergence between big data, spatial epidemiology, and health intelligence. Thus, the study offers an original contribution by helping to understand the geography of chronic diseases in one of the world's largest metropolitan regions.
